# Expression of Glycogen synthase kinase 3-β (GSK3-β) gene in azoospermic men

**Published:** 2014-05

**Authors:** Hamid Nazarian, Marefat Ghaffari Novin, Mohammad Reza Jalili, Reza Mirfakhraie, Mohammad Hassan Heidari, Seyed Jalil Hosseini, Mohsen Norouzian, Nahid Ehsani

**Affiliations:** 1 *Department of Biology and Anatomical Sciences, Faculty of Medicine, Shahid Beheshti University of Medical Sciences, Tehran, Iran.*; 2 *Infertility and Reproductive Health Research Center, Shahid Beheshti University of Medical Sciences, Tehran, Iran.*; 3 *Department of Medical Genetics, Faculty of Medicine, Shahid Beheshti University of Medical Sciences, Tehran, Iran.*

**Keywords:** *Azoospermia*, *Wnt signaling pathway*, *glycogen synthase kinase 3 beta*, *Spermatogenesis*

## Abstract

**Background:** The Wnt/β- The Wnt/β-catenin signaling pathway is involved in many developmental processes in both fetal and adult life; its abnormalities can lead to disorders including several types of cancers and malfunction of specific cells and tissues in both animals and humans. Its role in reproductive processes has been proven.

**Objective:** This study was designed to evaluate the expression of the key regulator of this signaling pathway GSK3-β and its presumed role in azoospermia.

**Materials and Methods: **WNT3a protein concentration and GSK3-β gene expression levels were measured and compared between two groups of infertile men. The test groups consisted of 10 patients with obstructive and 10 non-obstructive azoospermia. The control group was selected among healthy men after vasectomies that were willing to conceive a child using a testicular biopsy technique. Samples were obtained by testicular biopsy and screened for the most common mutations (84, 86 and 255) in the SRY region before analyzing. GSK3-β gene expression was assessed quantitatively by real time-PCR.

**Results: **The WNT3a protein concentration had no significant difference between the two test groups and controls. Expression of GSK3-β was down-regulated in non-obstructive azoospermia (3.10±0.19) compared with normal (7.12±0.39) and obstructive azoospermia (6.32±0.42) groups (p=0.001).

**Conclusion:** Down-regulation of GSK-3β may cause to non-obstructive azoospermia. Regulation and modification of GSK-3β gene expression by drugs could be used as a therapeutic solution.

## Introduction

According to the World Health Organization, the fertility rate is estimated to be 10-15%. In approximately 50% of the cases, the problem is in men (1). Azoospermia is one of male fertility disorders in which the sperm is not found in the seminal plasma after centrifugation (2). Some genetic factors cause to azoospermia; these factors are divided into groups namely: chromosomal abnormalities such as Klinefelter's syndrome and genetic disorders. One genetic and cellular factor that plays a key role in normal spermatogenesis is the Wingless-type MMTV integration (Wnt) signaling pathway (3). 

The Wnt/β-catenin signaling pathway controls many developmental processes during the entire lifetime where its disorders can cause a wide range of pathologic defects including cancer in both animals and humans. Wnt signaling is generally done in two ways: The canonical pathway or Wnt/β-catenin in which the conserved β-catenin accumulates in the nucleus and promotes the target genes majorly involved in the proliferation, polarity, fate and differentiation in embryonic cell development and in the adult (4). In cells with inactive Wnt signaling pathway (in absence of Wnt activating ligands) cytoplasmic β-catenin is phosphorylated by triple complex APC-Axin-GSK3-β, ubiquitinated and degraded by proteasomes. 

Consequently, β-catenin does not enter the nucleus and target genes will not be transcripted. The non-canonical pathway acts via activation of small GTPase Rho and Rac and causes rearrangement of the cytoskeleton and asymmetry of epithelial plates and other structures (5). In some cases, the non-canonical Wnt pathway can inhibit the canonical, e.g. competition for protein Disheveled (Dvl), which is shared between the two pathways and increased expression of Siah2 (siah E3 ubiquitin protein ligase 2) causes to degrade β-catenin by Wnt5a (5, 6). 

This signaling pathway is closely associated with kinases and transcription factors of nuclear receptors, including the androgen receptor (AR) and estrogen receptors (ERs) (7). Interaction between the Wnt signaling pathway androgen receptor is essential for normal prostate development; disorders cause cancer and tumor progression (8). Wnt maintains proliferation and stemness of human spermatogonial stem cells (SSCs) (9). Over activation of Wnt causes proliferation and inhibition of differentiation of Sertoli cells. Consequently, these cells do not support germ cells which lead to increased apoptosis and male infertility (10). 

Expression of Wnt proteins of several types including 1, 3, 4, 5a and 7a in the testes of fetal and adult humans and rodents has been shown (11-15). Additionally, the other components of the canonical pathway such as Fz9, Nkd1 and β-catenin an antagonist of this pathway has been identified in the testis (16). Given the importance of the Wnt signaling pathway in male reproduction especially spermatogenesis, this study was designed to evaluate the expression of GSK3-β the key regulator of this signaling pathway and its presumptive role in azoospermia.

## Materials and methods

**Sampling and preparation**

This observational, case-control study was conducted in 2013 after obtaining approval from the Ethics Committee of the faculty of medicine of Shahid Beheshti University of Medical Siences. The test groups consisted of 10 patients with obstructive and 10 non-obstructive azoospermia. Specimens were obtained by testicular sperm extraction (TESE). Samples were considered as obstructive azoospermic if spermatozoa were observed in the biopsy, otherwise as non-obstructive azoospermia. The control group was selected among healthy men after vasectomies that were willing to conceive a child using a testicular biopsy technique. Patients with Klinefelter's syndrome were excluded. Prior to the study Y chromosome microdeletions in patients with non-obstructive azoospermia and common mutations in the CFTR gene in patients with obstructive azoospermia were analyzed. 

All patients participating in the trial received information and signed a written consent. Testicular biopsy was performed by an urologist under sterile conditions and local anesthesia. Samples were placed in Dulbecco’s modified Eagle’s medium (DMEM) (Invitrogen, Carlsbad, U.S.A.) and were transferred to the laboratory within a maximum time of 1 hr.

**ELISA**

Equal volumes of tissue samples were put in 96 well plates with 100µL serum-free DMEM and incubated for 1h at 37⁰C while gently shaking in a Shaker incubator. Supernatant was aspirated entirely (100 µL) and the concentration of Wnt3a protein was measured using ELISA Kit for Wingless Type MMTV Integration Site Family, Member 3A (WNT3A) (USCN, Wuhan, China) according to the manufacturer’s instructions. Optical density (OD) of the samples was measured at 450 NM by Biotek ELX800 microplate reader (Biotek, Winooski, U.S.A.). Standard curves were plotted and Wnt3a concentrations were calculated against optical density (OD) and measured in pg/mL. Each test was repeated 3 times.

**Screening for frequent mutations using PCR**

After aspiration of the supernatant, DNA and RNA were extracted using Trizol solution (Invitrogen, Carlsbad, U.S.A.). Frequent mutations (SRY, 84, 86 and 255) were assessed using two separate Multiplex PCR according to the European Academy of Andrology (EAA) and the European Molecular Genetics Quality Network (EMQN) practice guidelines for molecular diagnosis of y-chromosomal microdeletions (Primers listed in Table I) (17). PCR reactions were performed using PCR master mix (Amplicon, Odense, Denmark) and Peqstar thermo cycler (Peqlab, Erlangen, Germany) devices. Reaction products (25 µL) were separated on a 2% Agarose (Invitrogen, Carlsbad, U.S.A.) gel in 1× TBE (Invitrogen, Carlsbad, U.S.A.) for 80V (1 hour).

**Real time-PCR**

Quantitative analysis of expression of the GSK3-β gene was carried out. Total RNA from the previous step was used as template. The standard reverse transcription was performed using Revert Aid First Strand cDNA Synthesis Kit (Thermo, Waltham, USA) according to the manufacturer’s instructions. GSK3-β primer (Forward: 5’-GGAACTCCAACAAGGGAGCA-3’, Reverse: 5’-TTCGGGGTCGGAAGACCTT A-3’) checked by RT-PCR in the aforementioned manner and reaction products (20 µL) were separated on a 1.5% Agarose gel in 1× for 80V (1 hour). Subsequent real time PCR was done with Power Syber Green real time master mix and Stepone real time PCR (Applied biosystems, Carlsbad, USA). GAPDH primer (Forward: 5’-ACAGTCAGCC GCATCTTCTT-3’, Reverse: 5’-ACGACCAAA TCCGTTGACTC-3’) was used as a housekeeping gene. Each reaction was performed in triplicate. 

**Statistical analysis**

The normality of data distribution confirmed by the Shapiro-wilk. The data were analyzed using one-way ANOVA, followed by Tukey test. Statistical analyses were performed using SPSS 14 software. P<0.05 was considered as significant level.

**Limitations**

Since the size of the biopsies was low, extraction of DNA and RNA from the same sample following ELISA assay was difficult. The number of available control samples was low as well.

**Table I:** Primers used for y-chromosomal microdeletions screening

**Primer**	**Sequence**	**Product size (bp)**
SRY-F	5’-GAATATTCCCGCTCTCCGGA-3’	472
SRY-R	5’-GCTGGTGCTCCATTCTTGAG-3’	
86-F	5’-GTGACACACAGACTATGCTTC-3’	320
86-R	5’-ACACACAGAGGGACAACCCT-3’	
84-F	5’-AGAAGGGTCTGAAAGCAGGT-3’	326
84-R	5’-GCCTACTACCTGGAGGCTTC-3’	
255-F	5’-GTTACAGGATTCGGCGTGAT-3’	126
255-R	5’-CTCGTCATGTGCAGCCAC-3’	

## Results

**Screening for frequent mutations**

**Figure 1:**
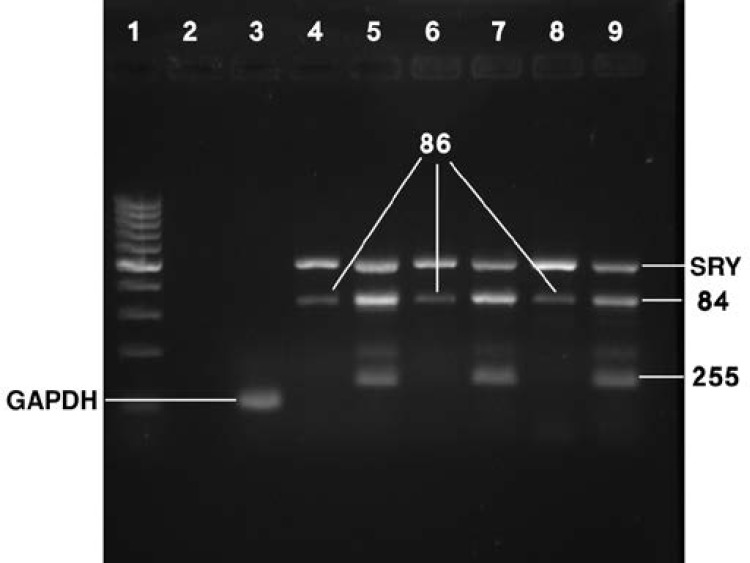
Screening for SRY, 84 and 86 mutations by multiplex PCR. 25 µL PCR Reactions were prepared according to the European Academy of Andrology (EAA) and the European Molecular Genetics Quality Network (EMQN) practice guidelines for molecular diagnosis of y-chromosomal microdeletions. PCR products were separated on 2% agarose gel pre stained with SYBR safe dye (Life technologies, NY, USA). Lane1: 100bp Gene ladder, Lane 2: Negative control, lane 3: Housekeeping gene, Lanes 4 and 5: sample 1, Lanes 6 and 7: Sample 2, Lanes 8 and 9: Sample 3

None of the samples had mutations in tested genes and all samples were studied. PCR results of 3 samples are shown in Figure 1.

**ELISA**

The results of ELISA showed that there were no significant differences in concentration of Wnt3a protein between samples from patients (Obstructive azoospermia: 46.069±2.69, Non-obstructive Azoospermia: 45.519±2.58) and healthy subjects (47.071±2.78) (p=0.505). In each group Wnt3a concentration of one sample was significantly different from the rest and was replaced with another new sample. 

**Real time-PCR**

Quantitative real-time RT-PCR analysis of normal (healthy), obstructive azoospermia and non-obstructive azoospermia testicular samples were performed for GSK3-β gene expression. The primers were previously checked by conventional RT-PCR and electrophoresis on 1.5% agarose gel (Figure 3). Expression of GSK3-β was down-regulated in non-obstructive azoospermia (3.10±0.19) compared to normal (7.12±0.39) and obstructive azoospermia (6.32±0.42) groups. The difference was significant (p=0.001) (Figure 3 and 4).

**Figure 2:**
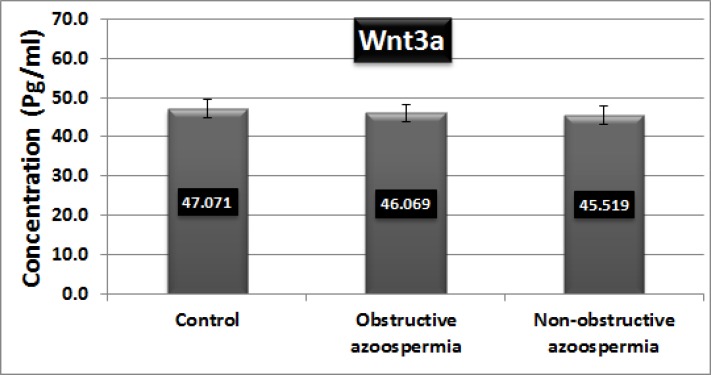
Wnt3a protein measured by a commercial Wnt3a ELISA Kit. Optical density (OD) of the samples was measured at 450 nm. Wnt3a concentrations were stated in pg/mL. There is no significant difference in concentration of Wnt3a protein between samples from patients and healthy subjects (Obstructive azoospermia: 46.069±2.69 and Non-obstructive Azoospermia: 45.519±2.58) and healthy subjects (47.071±2.78) (p=0.505).

**Figure 3:**
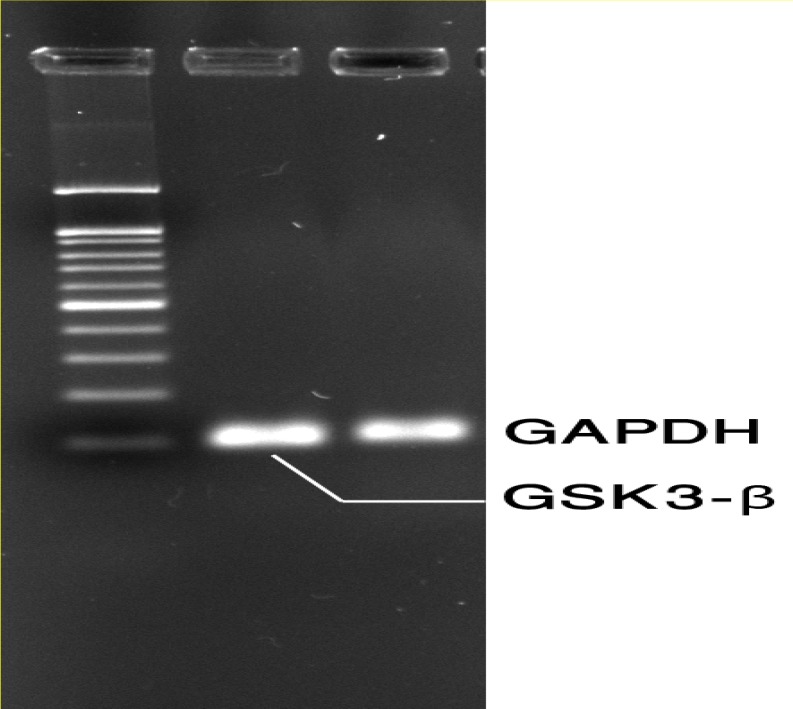
RT-PCR was performed with GAPDH and GSK3-β primers and products were separated by electrophoresis on 1.5% agarose gel pre stained with SYBR safe dye (Life Technologies, NY, U.S.A.); Lane1: 100bp Gene ladder, Lane 2:GSK3 and lane 3: Housekeeping gene (GAPDH).

**Figure 4:**
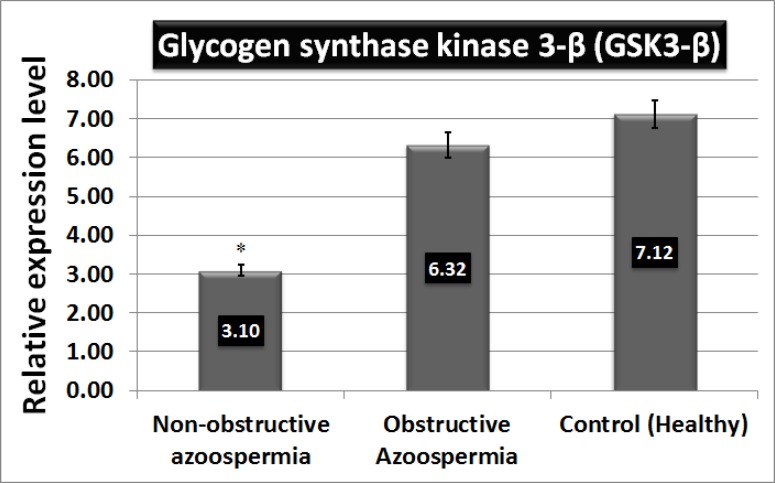
Real time-PCR for GSK3-β; Total RNA was extracted; the standard reverse transcription was performed using RevertAid First Strand cDNA Synthesis Kit. Subsequent real time PCR was done with Power SYBR Green real time master mix and Stepone real time PCR (Applied Biosystems, Carlsbad, U.S.A.). GAPDH primer was used as a housekeeping gene. Each reaction was performed in triplicate. Expression of GSK3-β was down-regulated in non-obstructive azoospermia (3.10±0.19) compared with normal (7.12±0.39) and obstructive azoospermia (6.32±0.42) groups. The difference was significant (p=0.001)

## Discussion

In this study the possible effects of Wnt pathway abnormalities in azoospermia were examined. Since 3-dimensional tissue structure and cell-cell and cell-extracellular matrix (ECM) interactions are highly involved in cellular responses and performance, we attempted to maintain this condition with as little change as possible for testing. With this aim, cultivation and maintenance of tissues in the laboratory was avoided. The restructuring of the tissue, isolation, proliferation and long-term storage of cells in culture media was prevented. Microdeletions of the Y chromosome are the second most frequent genetic cause of male infertility (17). Deletions lead to clinical abnormalities found in patients with azoospermia or sperm concentration <1×10^6^/mL. Infertile males with sperm concentration between 1 and 5×10^6^/mL can rarely have deletions (18, 19). 

Thus, their genetic diagnosis is routinely tested in azoospermic men. In this study patients were screened for the most common mutations (SRY, 84, 86 and 255) in the SRY region according to the European Academy of Andrology (EAA) and the European Molecular Genetics Quality Network (EMQN) practice guidelines for molecular diagnosis of y-chromosomal microdeletions (17). Wnt3a increases the proliferation of a spermatogonial cell line in vitro by activating β-catenin signaling pathway (9). Wnt3a protein the activator ligand of canonical Wnt/β-catenin signaling pathway was measured as an exclusion criterion. 

The aim of this measurement was to determine whether the amount of Wnt3a protein has a significant difference between patients and healthy samples. The importance and necessity of this evaluation was to investigate the gene expression of intracellular components of the Wnt signaling pathway apart from the extracellular factors (Wnt ligand). One of the benefits and achievements of this research was the distinction between extracellular and intracellular portions of the signaling pathway that has made it more accurate followed by more reasonable interpretation of the results. 

In previous studies judgments about different attributes of Wnt signaling pathway, including activity and expression levels of genes encoding pathway components was performed regardless of the expression levels and concentration of the Wnt ligand. Golestaneh *et al* reported that Wnt/β-catenin pathways, especially Wnt3a, may play an important role in the regulation of mouse and human spermatogonia. They also demonstrated that Wnt3a induces cell proliferation, morphological changes, and cell migration in C18-4 cells. However, Wnt3a concentration levels and its effect on Wnt pathway activity were not considered. It should be noted that the behavior of cells can vary in vitro and in vivo (9). 

Boyer *et al* demonstrated that Sustained Wnt/CTNNB1 signaling in Sertoli cells causes testicular degeneration and the formation of foci of poorly differentiated stromal cells in the seminiferous tubules in mice. In this study the concentration of Wnt protein was not measured, as well (20). GSK3-β, a protein kinase, phosphorylates and inactivates glycogen synthase and was discovered 20 years ago (21, 22). This protein is the key regulator of Wnt/β-catenin signaling pathway; its abnormalities cause many disorders in fetal development and adult growth and differentiation including genital organ cancers, germline incompetence, function of specialized cells and many other reproductive diseases often which lead to infertility (10). 

Thus, we studied the expression of GSK3-β gene as a main component of the Wnt/β-catenin signaling pathway measured quantitatively with real time-PCR. Results showed that gene expression was significantly down-regulated in non-obstructive azoospermic men. It is in agreement with previous studies that revealed that GSK3-β expression has a critical role in mice germ cell development and differentiation and its disorders result in testicular degeneration, testicular cord disruption and Mullerian duct regression (23, 24). Also, aberrant expression of β-catenin due to down-regulation of GSK3-β leads to abnormal development of primordial germ cells (25). 

On the other hand, inhibition of GSK-3β in cultured adult human Sertoli cells by its inhibitors (SB216763 and lithium chloride) activates Wnt/β-catenin signaling, induces an increase in c-Myc expression and cell proliferation (26). Most previous studies have been performed on mice and rats. Though wnt signaling pathway is conserved through evolution, its function in cells and tissues of either the same or different species can vary. Therefore, in this study Wnt signaling pathway in human testicular tissue was investigated that in the past has been less studied. 

Also as mentioned above, in this study, a judgment about the level of GSK3 gene expression and thus the activity of Wnt/β-Catenin pathway was done after measurement of concentration of Wnt3a protein as extracellular factors. This led to examine features and functions of investigated cells and tissue, regardless of endocrine and paracrine affecting factors.

## Conclusion

In summary, Wnt3a concentration has no significant difference in azoospermic men compared with the control group. GSK-3β was down-regulated significantly in non-obstructive azoopermic men. 
